# Immunohistochemistry using an antibody to unphosphorylated connexin 43 to identify human myometrial interstitial cells

**DOI:** 10.1186/1477-7827-6-43

**Published:** 2008-09-16

**Authors:** Graham Hutchings, Thomas Gevaert, Jan Deprest, Tania Roskams, Alfons Van Lommel, Bernd Nilius, Dirk De Ridder

**Affiliations:** 1 Department of Obstetrics and Gynaecology, University Hospital Gasthuisberg, Katholieke Universiteit Leuven, Herestraat 49, B-3000 Leuven, Belgium; 2 Department of Morphology and Molecular Pathology, University Hospital Gasthuisberg, Katholieke Universiteit Leuven, Herestraat 49, B-3000 Leuven, Belgium; 3 Department of Physiology, University Hospital Gasthuisberg, Katholieke Universiteit Leuven, Herestraat 49, B-3000 Leuven, Belgium; 4 Department of Urology, University Hospital Gasthuisberg, Katholieke Universiteit Leuven, Herestraat 49, B-3000 Leuven, Belgium

## Abstract

**Background:**

Myometrial smooth myocytes contract as a result of electrical signalling via a process called excitation-contraction coupling. This process is understood in great detail at the cellular level but the generation and coordination of electrical signals throughout the myometrium are incompletely understood. Recent evidence concerning the vital role of interstitial cells of Cajal in tissue-level signalling in gastrointestinal tract, and the presence of similar cells in urinary tract smooth muscle may be relevant for future research into myometrial contractility but there remains a lack of evidence regarding these cells in the myometrium.

**Methods:**

Single stain immunohistochemical and double stain immunofluorescence techniques visualised antibodies directed against total connexin 43, unphosphorylated connexin 43, KIT, alpha-SMA and prolyl 4-hydroxylase in myometrial biopsies from 26 women representing all stages of reproductive life.

**Results:**

Myometrial smooth myocytes from term uterine biopsies expressed connexin 43 in a punctate pattern typical of gap junctions. However, on the boundaries of the smooth muscle bundles, cells were present with a more uniform staining pattern. These cells continued to possess the same staining characteristics in non-pregnant biopsies whereas the smooth myocytes no longer expressed connexin 43. Immunohistochemistry using an antibody directed against connexin 43 unphosphorylated at serine 368 showed that it is this isoform that is expressed continually by these cells. Double-stain immunofluorescence for unphosphorylated connexin 43 and KIT, an established marker for interstitial cells, revealed a complete match indicating these cells are myometrial interstitial cells (MICs). MICs had elongated cell processes and were located mainly on the surface of the smooth muscle bundles and within the fibromuscular septum. No particular arrangement of cells as plexuses was observed. Antibody to prolyl 4-hydroxylase identified fibroblasts as separate from MICs.

**Conclusion:**

MICs are identified consistently on the boundaries of smooth muscle bundles in both the pregnant and non-pregnant uterus and are distinct from fibroblasts. The uniform distribution of connexin 43 on the cell membrane of MICs, rather than localisation in gap junction plaques, may represent the presence of connexin hemichannels. This antibody specificity may aid future study of this potentially important cell type.

## Background

Although the cellular processes governing the contractility of individual myocytes are understood in great detail, the mechanism for the coordination of uterine activity is less well understood [1,2]. It is observed that connexin 43 expression is greatly increased at the time of parturition and it has been proposed that this connexin, in the form of gap junctions, leads to the formation of a 'functional syncytium' providing for the coordination of contractions throughout the myometrium [3]. However this model does not explain a mechanism for pacemaking and may not be compatible with the bell-shaped contraction profile observed [4]. Also it does not explain the existence of the coordinated uterine activity that is observed when connexin expression is low, such as during pregnancy before labour, during the menstrual cycle [5] or around the time of fertilisation [6], (the coordinated uterine activity at these times implies the presence of a permanent mechanism for generating uterine contractions).

In the gastrointestinal tract, interstitial cells of Cajal have been shown to be vital in coordinating pacemaking and signal transduction [7], whilst the smooth muscle cells in these tissues are responsible only for generating the contractile forces. Myometrial interstitial cells (MICs) have earlier been demonstrated [8,9] but their spatial relationships and their role in controlling uterine activity have not been defined.

This paper describes the topographical distribution of MICs by using single stain immunohistochemistry and double stain indirect immunofluorescence techniques with a combination of five antibodies; unphosphorylated connexin 43 (binds only when the serine at residue 368 is unphosphorylated), total connexin 43 (independent of phosphorylation status), KIT (a membrane bound tyrosine kinase receptor found on interstitial cells of Cajal) [10], alpha smooth muscle actin (a smooth muscle marker), and prolyl 4-hydroxylase (a specific marker for fibroblasts) [11]. Further, it is proposed that the staining characteristics observed with the antibody for unphosphorylated connexin 43 represent the presence of connexin hemichannels rather than gap junctions and that these hemichannels could be involved in cell-cell signalling.

## Methods

Myometrial biopsies were obtained from a total of 26 women. Six of the women were not pregnant, three premenopausal and three postmenopausal, all of whom were undergoing hysterectomy for benign disease. For continuity the site of biopsy was close to the right cornu. The remaining twenty samples from pregnant women were removed from the upper border of the lower segment incision at the time of caesarean section. Nine of these were obtained at the time of elective (pre-labour) caesarean at term and ten were obtained at the time of emergency caesarean section during active labour. The one other specimen was taken from the upper segment of a woman delivered at 27 weeks by classical caesarean section due to placental abruption. All women consented to the procedure and the Ethical Committee of the University Hospitals Leuven approved the clinical protocol.

All biopsies were snap frozen immediately in isopentane cooled with liquid nitrogen and underwent single stain immunohistochemistry. Sections measuring 5 μm in thickness were cut with an HM 560 Cryo-star Cryostat (MICROM International GmbH, Walldorf, Germany) and air-dried overnight before fixing for ten minutes in acetone. Monoclonal mouse anti-human antibody to unphosphorylated connexin 43 (Cat no: 13-8300, dilution 1/100, Zymed, San Francisco, California) was added for 30 minutes and rinsed with phosphate buffered saline (PBS) before adding polyclonal rabbit anti-mouse biotinylated immunoglobulin (dilution 1/400, DakoCytomation, Heverlee, Belgium). The slide was rinsed once again before adding Avidine-Biotinylated horseradish peroxidase complex for 30 minutes (ABComplex/HRP, DakoCytomation). The complex was stained with aminoethylcarbazole for ten minutes and the slide counterstained with Maeyers haematoxylin for 1 minute. A similar procedure was employed for all biopsies using a polyclonal antibody to total connexin 43 (Dilution 1/20, Sigma-Aldrich, Gillingham, UK), KIT (1/50, polyclonal rabbit antibody, DakoCytomation), and prolyl 4-hydroxylase (1/50, Abcam, Cambridge, UK). All slides were compared with controls in which the primary antibody had been omitted. Microscopy was performed with an Axioplan 2 upright microscope with image capture by Axiovision software (Carl Zeiss, Oberkochen, Germany).

Double-stain indirect immunofluorescence was performed on four myometrial biopsies, one from each of the above groups (premenopausal, postmenopausal, elective caesarean prior to the onset of labour and emergency caesarean during labour). Cryostat sections of a thickness of 10 μm (HM 560 Cryo-star) were dried overnight and fixed in acetone for ten minutes. Slides were labelled in sequence with monoclonal mouse anti-human antibody to unphosphorylated connexin 43 (dilution 1/100, Zymed), FITC-labelled rabbit anti-mouse antibody (FITC = fluoresceine isothiocyanate, dilution 1/20, DakoCytomation), mouse anti-human antibody to alpha smooth muscle actin (α-SMA, dilution1/40, DakoCytomation) and TRITc-labelled rabbit anti-mouse antibody (TRITc = tetramethylrhodamineisothiocyanate, dilution1/20, DakoCytomation). The duration of staining for each antibody was 1 hour and the slide was rinsed between each step with PBS. Finally the slide was mounted with glycerol buffer with the anti-fading agent PPD (p-Phenylenediamine DakoCytomation) before being sealed with nail polish.

A similar double staining procedure was performed on slides from the same biopsies using antibodies to unphosphorylated connexin 43 and KIT (Dilution 1/20). Again, controls consisted of the same steps with omission of one or both of the primary antibodies.

The slides were examined with a confocal laser-scanning microscope (LSM 410 Invert, Zeiss, Oberkochen, Germany) using either a ×40 or ×100 objective and electronic zoom. For each slide either a single section or a stack of five optical sections at one-micrometer intervals were digitally recorded for red and green fluorescence and stored on separate channels. Where multiple sections were recorded, these were superimposed using the extended depth of focus function. The coloured images were then overlaid to produce the final image.

## Results

**Figure 1:**
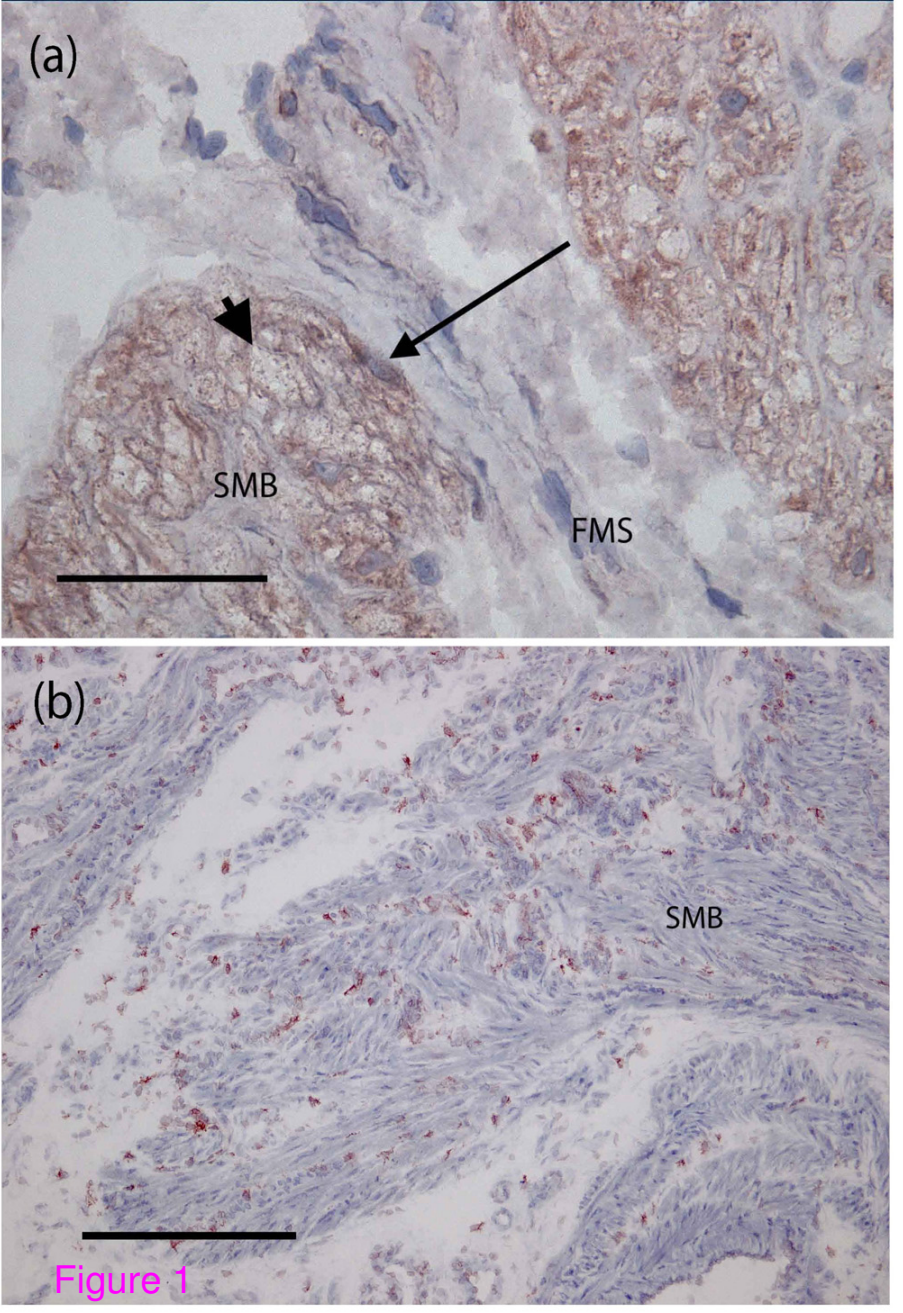
**Total connexin 43 distribution in myometrium during term labour**. (a) Peripheral punctate staining typical of gap junctions is seen on smooth myocytes (arrowhead). On the boundary of the smooth muscle bundles are cells with a homogenous staining pattern (arrow) (magnification ×20, Bar 50 micrometres, SMB = smooth muscle bundle, FMS = fibromuscular septum). (b) The cells on the boundary of the smooth muscle bundles exhibit the same pattern of staining in the non-pregnant myometrium. In contrast the smooth myocytes do not exhibit the punctate staining seen in the biopsy obtained during term labour (magnification ×10, Bar 100 micrometres, SMB = smooth muscle bundle).

Single stain immunohistochemistry for total connexin 43 in all biopsies from labouring women was positive for smooth muscle cells within the muscle bundles with peripheral punctate staining of the cytoplasm typical of gap junctions (Figure 1a). In addition, positive cells with elongated cell bodies were seen on the boundaries of the smooth muscle bundles and cells of similar shape were also apparent within the fibromuscular septum. The antibody binding to these cells appeared homogenous throughout the cell membrane in contrast to the distribution observed on smooth myocytes. A similar picture was evident in all the biopsies from pre-labour women but in biopsies from non-pregnant women the smooth myocytes did not bind the antibody. However the cells on the boundaries of smooth muscle bundles and within the fibromuscular septum expressed connexin 43 consistently in every biopsy indicating that this expression does alter with the occurrence of pregnancy or the development of labour (Figure 1b).

**Figure 2:**
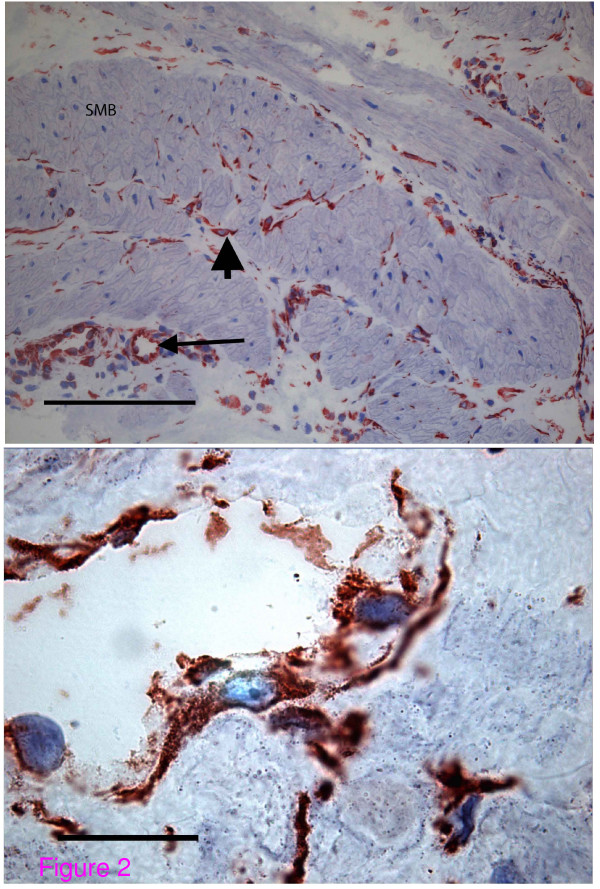
**Unphosphorylated connexin 43 distribution in myometrium during term labour**. (a) Antibody for unphosphorylated (serine 368) connexin 43 binds only to cells on the boundaries of the smooth muscle bundles (arrowhead) and to vascular endothelial cells (arrows) but not to smooth myocytes (Magnification ×10, Bar 100 micrometres). (b) At higher magnification, these cells show homogenous staining throughout the cell membrane, an oval shaped nucleus, elongated cell body and extended dendritic processes. (Magnification ×100, Bar 10 micrometres).

In contrast to total connexin 43, unphosphorylated connexin 43 was confined to the cells described above, located mainly on the boundary of the smooth muscle bundles and in the fibromuscular septum in all of the biopsies examined (Figure 2a). In all biopsies from pregnant women in labour or prior to labour, the antibody for unphosphorylated connexin 43 did not bind to the smooth myocytes. Under higher-powered magnification the positive cells had oval shaped nuclei and little perinuclear cytoplasm. The cell body was elongated and possessed dendritic processes (Figure 2b). Again the antibody was bound to the entire cell surface in contrast to the punctate staining seen on smooth myocytes with antibody to total connexin 43. Endothelial cells lining small blood vessels also expressed unphosphorylated connexin 43 but were distinguishable by their continuity with other similar cells to form vessels. Examination of the single biopsy taken from the upper segment of the uterus revealed an identical pattern. In biopsies taken from non-pregnant pre-menopausal women the smooth muscle bundles showed less hypertrophy making rows of unphosphorylated connexin 43 expressing cells more easily visible (data not shown). Although this architecture was less apparent in samples from postmenopausal women, unphosphorylated connexin 43 expressing cells were still identifiable as separate from smooth muscle cells that did not express connexin 43 (data not shown). Thus all biopsies demonstrated the permanent presence of unphosphorylated connexin 43-expressing cells on the boundaries of the smooth muscle bundles.

**Figure 3:**
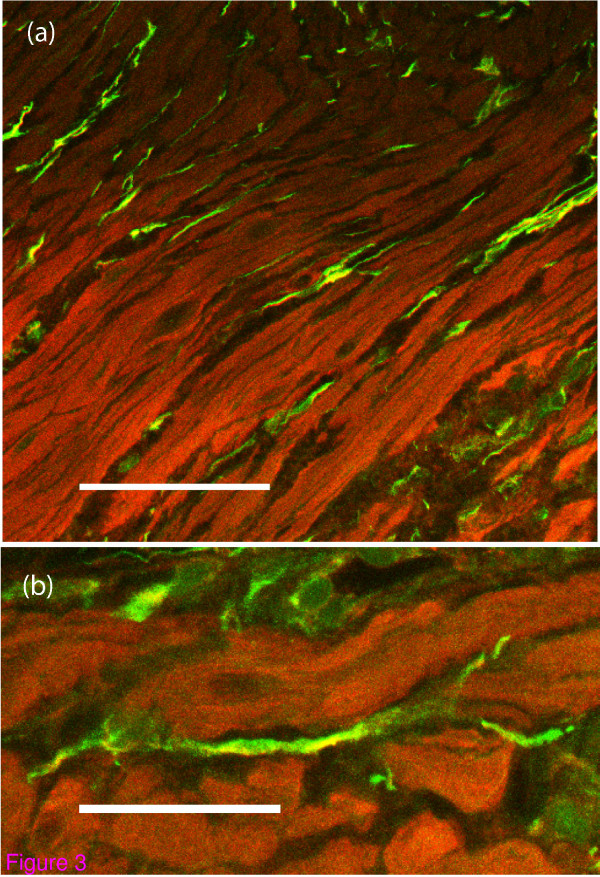
**Double stain immunofluorescence for unphosphorylated connexin 43 and alpha smooth muscle actin**. (a) Double stain immunofluorescence using FITC labelled unphosphorylated connexin 43 (green) and TRITc labelled alpha SMA (red). Again, cells expressing unphosphorylated connexin 43 are identified on the boundaries of smooth muscle bundles. The Myometrial biopsy is from a woman in spontaneous labour at term (Bar = 50 micrometres). (b) A higher-powered view of the same biopsy from a woman in spontaneous labour at term showing dendritic processes reaching between the myocytes. The antibody is bound evenly to the entire surface of the cell (Bar = 10 micrometres).

Immunofluorescence provides crisp clear images for a more precise localisation of bound antigens and was used to confirm the homogenous pattern of binding of unphosphorylated connexin 43 found with the HRP method. Examination of the double-stained immunofluorescence slides stained for α-SMA and unphosphorylated connexin 43 confirmed the appearances of the standard single stain immunohistochemistry slides (Figure 3a and Figure 3b). Smooth muscle bundle architecture was visualised clearly with the antibody to α-SMA. The antibody to unphosphorylated connexin 43 again demonstrated homogenous binding to the entire cell membrane and showed a population of cells arranged longitudinally within the connective tissue septa. Dendritic processes were seen, some of which reached in between the smooth muscle cells and some of which could be followed along their length towards those of other similar cells. The same topographical appearances were observed throughout the entire thickness of the myometrium including that of the sample from the upper segment. No areas of increased cellular density constituting what could be described as a plexus was identifiable at any region of the myometrium.

**Figure 4:**
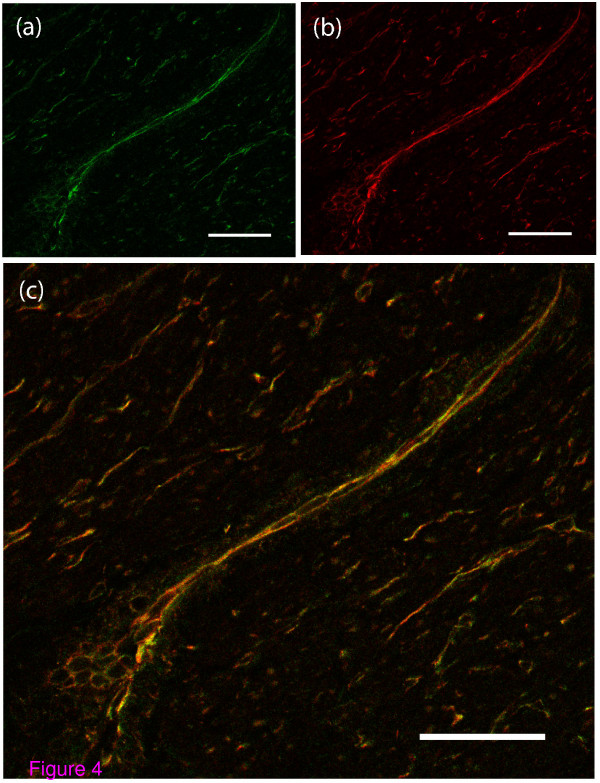
**Double stain immunofluorescence for unphosphorylated connexin 43 and c-kit in myometrium during term labour**. Immunofluorescence using (a) FITC labelled unphosphorylated connexin 43 (Green), (b) TRITc labelled KIT antibodies (Red) and (c) overlap of the images to reveal a complete match indicating that the unphosphorylated connexin 43 expressing cells also express KIT (Bars = 150 micrometres). This finding indicates that these cells are MICs. The distribution of KIT is even throughout the entire cell membrane.

Double stain immunohistochemistry using anti-KIT antibody (KIT is a marker of interstitial cells of Cajal in the gut), and unphosphorylated connexin 43 antibody was performed. Superimposing the immunofluorescence patterns obtained with both antibodies showed a perfect match indicating co-expression of these two proteins in these cells (Figure 4a, Figure 4b and Figure 4c). KIT was distributed evenly throughout the entire cell membrane. Controls where one or other of the primary antibodies was omitted confirmed that this was genuine co-localisation. This indicates that the cells that bind unphosphorylated connexin 43 are likely to be MICs.

**Figure 5:**
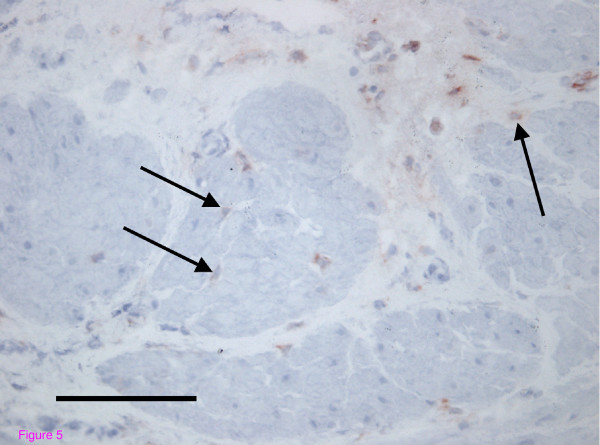
**Immunohistochemistry for fibroblasts using an antibody for prolyl 4-hydroxylase in myometrium during term labour**. Prolyl 4-hydroxylase is an enzyme critical to the production of collagen and its presence is used as a marker for fibroblasts. Immunohistochemistry for this enzyme showed fibroblasts (arrows) in fewer numbers than the cells identified by unphosphorylated connexin 43 staining. These fibroblasts were also distributed more evenly throughout the myometrium and were located more frequently within the smooth muscle bundles rather than on the surface of the smooth muscle bundles (Magnification × 20, Bar 50 micrometres).

Staining for prolyl 4-hydroxylase showed the presence of fibroblasts in fewer numbers and with a more even distribution throughout the myometrium when compared with MICs showing that these are two distinct populations of cells (Figure 5).

## Discussion

The appearance, number and location of the cells that we identified by staining for unphosphorylated connexin 43 and c-kit correspond most probably with MICs. Previously, MICs containing numerous mitochondria have been identified in the uterus and account for approximately 10% of the total number of cells in the myometrium [8]. In addition, cell-cell contact via gap junctions between adjacent interstitial cells and with smooth myocytes has been shown previously [9]. From our observations it can be seen that these cells are numerous and appear to be arranged mostly on the surface of the smooth muscle bundles but also occasionally as discrete cells within the smooth muscle bundles themselves. Although similar in size and shape to fibroblasts, they can be distinguished by immunohistochemistry according to the expression of both unphosphorylated connexin 43 and KIT and by a lack of expression of prolyl 4-hydroxlase. The topographical arrangement of MICs is similar to that of interstitial cells found in the detrusor muscle of the bladder where interstitial cells are arranged along the boundary of smooth muscle bundles and in networks through the fibromuscular septum [12]. However in the bladder, interstitial cells are also concentrated beneath the urothelium to form a suburothelial plexus and are closely related to nerves. In the uterus no such plexuses were identified and nerves identified by immunostaining to β-3-tubuline were sparse and did not appear related to the interstitial cell network (data not shown). These structural differences are likely to reflect the fact that the bladder is a sensory organ as well as a contractile organ.

It is important to note that with the exception of one upper segment biopsy, all the samples in our study obtained from pregnant women were from the lower uterine segment. In other species such as rodents, the uterus is a tubular structure and effective labour requires peristalsis that starts at the corn of the uterus for the expulsion of the fetuses. In theory, the study of a pacemaking mechanism in such a system may be more informative if it were to use myometrium obtained from the corn of the uterus. In humans, however, the uterus is more globular in form. Given this shape, it has been argued that pressure generation is more important than peristalsis for the expulsion of uterine contents [13]. In addition to this no specific pacemaking area has been identified anatomically or functionally in the upper segment. It is also recognised that strips of muscle from the lower segment possess autonomous contractility (indicative of the presence of a pacemaking mechanism in muscle from this region), and biopsies from this site are used frequently for in vitro contractility studies. These points suggest the presence of a generalised pacemaking system throughout the myometrium and given the additional fact that upper segment biopsies are more difficult to obtain in clinical practice, it is reasonable use lower segment biopsies for the study of myometrial pacemaking. It should also be said that the immunohistochemistry appearances of the single upper segment biopsy in pregnancy and those from non-pregnant subjects were similar to those of the lower segment biopsies.

Concerning the coordination of myometrial smooth muscle contractility, it is thought that the smooth myocytes become coupled electrically at the time of labour by connexin 43 gap junctions to form a functional syncytium [3]. There is a large amount of supportive evidence related to this showing increased levels of connexin 43 protein and increased numbers of gap junctions on the smooth myocyte cell membrane associated with parturition. These gap junctions are thought to be essential for the electrical signalling that provides the stimulus for uterine contractions. At times other than labour, it is thought that the lack of gap junctions prevents electrical signalling and maintains uterine quiescence. This theory has remained essentially unchanged for the last 30 years but despite being widely accepted, it fails to explain adequately the presence of uterine contractions of similar profile before labour [14], of non-gravid uterine contractions [5,6], and does not provide an explanation for action potential generation (pacemaking). In the gastrointestinal tract, interstitial cells of Cajal act as signal transducers and pacemakers. If MICs in the uterus were to act in the same way then they may provide a mechanism to account for pacemaking and uterine contractility throughout reproductive life. The well-documented presence of gap junctions at the time of parturition could then facilitate the transmission of cell-cell signals through the smooth muscle bundles.

Our findings with antibody directed against total connexin 43 are consistent with the increased expression of connexin 43 in the myometrium around the time of labour although it can also be seen that connexin 43 is also present at times other than labour. Using the specific antibody directed against unphosphorylated connexin 43, we have shown that it is MICs and not smooth myocytes that continually express this connexin 43, and that this connexin 43 is distributed evenly throughout the MIC cell membrane in its unphosphorylated form. The antibody used has had its specificity for unphosphorylated connexin 43 tested extensively in the field of brain and cardiac research [15-17]. The even distribution of unphosphorylated connexin 43, rather than being localised at points only where cell-to-cell contact via gap junctions is possible, may be important when considering the function of MIC. Although identification of connexin 43 is often considered to equate to the presence of gap junctions, the connexin is first transported to the entire cell membrane in the form of connexon 'hemichannels' [18,19]. Essentially these hemichannels are connexin hexamers that have not docked with a connexin hexamer of a neighbouring cell as during the formation of a gap junction. It is unclear as to whether all of these eventually become incorporated into gap junctions but it is clear that whilst in their un-docked state on the cell membrane they are functionally active [20,21]. They have been shown to be involved with cell-cell signalling between astrocytes via calcium waves using mediators such as ATP [22], they can open in response to membrane depolarisation [20] and their permeability can be changed, for example by products of arachidonic acid metabolism [23]. Of particular interest, hemichannel function appears to be controlled by protein kinase C dependant phosphorylation of connexin 43 at serine residue 368 with the unphosphorylated state resulting in increased permeability [24]. The antibody we used specifically binds to connexin 43 only when this particular serine is unphosphorylated. Therefore we propose that a possible explanation for the even distribution of unphosphorylated connexin 43 on MICs may be that this represents the presence of functionally active connexin 43 hemichannels rather than gap junctions. These hemichannels may be involved in cell-to-cell signalling between MICs and smooth myocytes within the myometrium. Interestingly, our research group has observed that extracellular ATP is indeed active in controlling the frequency of myometrial contractility (unpublished data), and the release mechanisms for this extracellular ATP, including the possibility of functional connexin hemichannels in the myometrium, are presently being studied.

## Conclusion

In summary, the finding of MICs on the boundaries of uterine smooth muscle bundles in myometrial biopsies taken throughout reproductive life may have important implications. Interstitial cells in both the gut and the bladder are believed to be critical to the processes for contractile signal initiation and coordination. MICs have the potential to perform a similar role in the myometrium and further study of these cells may improve our future understanding of uterine contractility.

## Competing interests

The authors declare that they have no competing interests.

## Authors' contributions

GH, TG, JD and DDR conceived the study and participated in its design. JD took the myometrial biopsies. GH and TR imaged and interpreted the immunohistochemistry and GH and AVL imaged and interpreted the immunofluorescence. GH drafted the manuscript and BN revised the manuscript critically. All authors read and approved the final manuscript.
